# Health related quality of life among myocardial infarction survivors in the United States: a propensity score matched analysis

**DOI:** 10.1186/s12955-017-0809-3

**Published:** 2017-12-04

**Authors:** Lea Mollon, Sandipan Bhattacharjee

**Affiliations:** Department of Pharmacy Practice and Science, College of Pharmacy, The University of Arizona, 1295 North Martin Avenue, Tucson, AZ 85721 USA

**Keywords:** Health-related quality of life, Myocardial infarction, Propensity scores, Patient-reported outcomes

## Abstract

**Background:**

Little is known regarding the health-related quality of life among myocardial infarction (MI) survivors in the United States. The purpose of this population-based study was to identify differences in health-related quality of life domains between MI survivors and propensity score matched controls.

**Methods:**

This retrospective, cross-sectional matched case-control study examined differences in health-related quality of life (HRQoL) among MI survivors of myocardial infarction compared to propensity score matched controls using data from the 2015 Behavioral Risk Factor Surveillance System (BRFSS) survey. Propensity scores were generated via logistic regression for MI survivors and controls based on gender, race/ethnicity, age, body mass index (BMI), smoking status, and comorbidities. Chi-square tests were used to compare differences between MI survivors to controls for demographic variables. A multivariate analysis of HRQoL domains estimated odds ratios. Life satisfaction, sleep quality, and activity limitations were estimated using binary logistic regression. Social support, perceived general health, perceived physical health, and perceived mental health were estimated using multinomial logistic regression. Significance was set at *p* < 0.05.

**Results:**

The final sample consisted of 16,729 MI survivors matched to 50,187 controls (*n* = 66,916). Survivors were approximately 2.7 times more likely to report fair/poor general health compared to control (AOR = 2.72, 95% CI: 2.43–3.05) and 1.5 times more likely to report limitations to daily activities (AOR = 1.46, 95% CI: 1.34–1.59). Survivors were more likely to report poor physical health >15 days in the month (AOR = 1.63, 95% CI: 1.46–1.83) and poor mental health >15 days in the month (AOR = 1.25, 95% CI: 1.07–1.46) compared to matched controls. There was no difference in survivors compared to controls in level of emotional support (rarely/never: AOR = 0.75, 95% CI: 0.48–1.18; sometimes: AOR = 0.73, 95% CI: 0.41–1.28), hours of recommended sleep (AOR = 1.14, 95% CI: 0.94–1.38), or life satisfaction (AOR = 1.62, 95% CI: 0.99–2.63).

**Conclusion:**

MI survivors experienced lower HRQoL on domains of general health, physical health, daily activity, and mental health compared to the general population.

## Background

Each year, approximately 735,000 people in the United States (U.S.) experience a myocardial infarction (MI) making it one of the leading causes of death and disability. Of these, 525,000 are new attacks and 210,000 are recurrent attacks [1]. Complications after MI are well documented and include a higher risk of additional cardiovascular events such as heart failure, angina, arrhythmias, stroke, or death [2]. With the advent of reperfusion and preventative therapies, short-term mortality following MI has decreased [1]. Consequently, longer survival post-MI has led to a growing group of individuals at an increased risk for complications impacting quality of life. An existing study conducted in Germany demonstrated that health-related quality of life (HRQoL) is significantly decreased in MI survivors compared to the general population [3]. Another study by Mendes de Leon et al. conducted in 1998 with a sample of older adults from Connecticut showed decrease in psychological, physical, and social functioning, especially among older adults [4]. A review by Mierzyńska et, al. also underscored the psychological burden experienced following MI outlining the importance of social support [5]. Furthermore, a review conducted in 2003 showed that work status, physical capacity, functional status, symptoms, and general health perceptions decline after an MI [6]. A study conducted among a Canadian population demonstrated that differences in HRQoL were pronounced among those with comorbidities, such as diabetes [7]. Additionally, MI survivors are more likely to report lower HRQoL outcomes compared to individuals suffering from other chronic conditions [8]. With an aging population in the U.S. and people living longer, even after experiencing MI, HRQoL has become an important measure related to short- and long-term recovery and has prognostic value [9]. Importantly, assessing HRQoL has value in determining which individual characteristics are related to reduced HRQoL so that clinicians can identify possible interventions to relieve symptoms, prolong life, improve functionality, and increase participation in activities of daily living [10].

Studies investigating HRQoL among MI survivors compared to the general population are limited, with a focus on surgical populations [11], comorbid cardiac risk factors [12], or the influence of social factors [13]. Furthermore, existing studies are mostly conducted in an international setting [3, 14, 15], are older [4], lack generalizability [11], and did not account for selection bias [3]. The aim of this study was to determine if there were differences in HRQoL domains in MI survivors compared to propensity score matched controls in a nationally representation U.S. sample using the most recently available (2015) Behavioral Risk Factor Surveillance System (BRFSS) data. The study sought to identify which HRQoL domains (general health, activity limitation, physical health, mental health, social support, sleep, life satisfaction) differed between the two groups once matched on variables of age, gender, race/ethnicity, smoking status, BMI, and comorbidities.

## Methods

### Study design

This study used a retrospective, cross-sectional matched case-control study design. HRQoL among MI survivors was compared to a matched non-MI sample. Observational studies have identified several patient characteristics that are predictors of reduced HRQoL following a MI: age [16, 17], race [18], gender [3, 19, 20], body mass index (BMI) [3], smoking status [3] and presence of comorbidities [11, 12, 21]. Therefore, in this study, survivors were matched against those without a prior MI on age, gender, race and ethnicity, smoking status, BMI, and comorbidities (cancer, arthritis, stroke, diabetes, hypertension, depression, and asthma) to minimize the effect of selection bias and make these two groups comparable on observable characteristics.

### Data

MI survivors and controls were derived utilizing data from the 2015 BRFSS survey. The total sample size for the 2015 BRFSS was 441,456.

The BRFSS is a system of health-related telephone surveys administered annually by the Centers for Disease Control and Prevention (CDC) to non-institutionalized adults aged 18 and older in all 50 states, the District of Columbia, and three U.S. territories (e.g., US Virgin Islands, Guam, and Puerto Rico). The survey utilizes random digit dialing to landline and cellular phone lines for sample collection. Close to 500,000 interviews are performed annually making the survey the largest health survey system in the world. It provides self-reported data on disease prevalence, chronic health conditions, health-related risky behaviors, preventive health care utilization, self-perceived health status, access to health care services, use of preventative services, and sociodemographic and environmental characteristics. To maintain consistency, the BRFSS has standard protocols for data collection that consist of core questions on quality of life [22]. Although the BRFSS does not contain an MI-specific health status tool, such as the Seattle Angina Questionnaire (SAQ), it has been validated against existing HRQoL instruments, such as the Short Form 36 (SF-36), and found to be valid in terms of construct, criterion, and known-groups [23]. Additionally, prior studies have repeatedly tested the BRFSS for validity and reliability and found to be reliable and have a high overall validity [24]. Further details are available elsewhere [22].

### Study sample

The study sample consisted of older adults aged 50 or older who completed the BRFSS. MI survivors were identified by indicating a “yes” response to the question: “Has a doctor, nurse, or other health professional ever told you that you had a heart attack, also called a myocardial infarction?” The total sample consisted of 25,472 MI survivors. Of those, 16,729 met study inclusion criteria. MI survivors were matched 1:3 to non-MI controls using the greedy algorithm with 8 to 1 digit matching. The algorithm matches the first control to a case with the most similar propensity score on the first 8 digits of the propensity score. Prior to matching, the sample of non-myocardial controls was 213,412.

### Measures

#### Dependent variables

The framework for the outcomes of this study was based on Wilson and Cleary’s Health-Related Quality of Life Model [25]. This model states that health measures are on a continuum of biological, social, and psychological complexity that can be described in five domains: biological/physiological factors, symptoms, functioning, general health perception, and overall quality of life. That is, there is a relationship between health measures and individual characteristics. An important aspect to understanding this linkage is general health perception, which has been shown to be an independent predictor of use of medical or mental health services and mortality [25]. Using this model, HRQoL measures were defined as: (1) perceived physical health defined as the number of days physical health was not good during the past 30 days (none, 1–15 days, 16–30 days); (2) perceived mental health defined as the number of days mental health was not good during the past 30 days (none, 1–15 days, 16–30 days); (3) activity limitations (yes or no) because of physical, mental, or emotional problems; (4) recommended sleep defined as less than 8 h of sleep in a 24 h period (yes or no); (5) emotional support defined as how often social support received (always/usually, sometimes, rarely/never); (6) life satisfaction (very satisfied, satisfied, dissatisfied, or very dissatisfied); and (7) perceived general health (excellent/very good, good, fair/poor).These health measures have been similarly categorized in prior literature with a similar with a comparable study design [26]. In our study, each outcome was chosen based on its representation of HRQoL domains: symptoms status, functional status, general health perceptions, social and psychological support, and life satisfaction. Perceived physical health and recommended sleep represent symptoms status, activity limitations represent functional status, emotional support represents social and psychological support, perceived mental health and perceived general health represent general health perceptions, and life satisfaction represents overall quality of life.

### Other variables

Additional determinants of health are marital status, income, education, employment status, insurance status, access to usual care, region, and rural versus urban location [27]. These characteristics of environment can also influence symptom or functional status, general health perceptions, and overall quality of life. Therefore, these variables were included in the analysis and defined as: (1) marital status (Married, Widowed, Sep/Divorced, Never Married); (2) education (less than high school, high school graduate, some college or technical school, college or technical school graduate); (3) employment status (employed, unemployed); (5) annual family income (less than 25 K, 25-35 K, 35-50 K, 50-75 K, greater than 75 K); (6) insurance status (insured, not insured); (7) usual source of care (yes or no); (8) region of the U.S. (Northeast, Midwest, South, West); and (9) metropolitan living status (Metropolitan living, non-metropolitan living).

### Statistical methods

Chi-square tests were performed to compare differences between MI survivors to controls on all variables before and after matching. HRQoL outcomes were estimated using binary logistic regression (life satisfaction, sleep quality, and activity limitations) and multinomial logistic regression (social support, perceived general health, perceived physical health, and perceived mental health). A-priori significance was set at *p* < 0.05. All analyses were conducted using SAS 9.4 (SAS Institute Inc.) using survey procedures that account for the complex survey design of the BRFSS. The study was conducted using de-identified, secondary data and met the University of Arizona Internal Review Board requirements for exempt non-human subject research.

## Results

**Fig. 1:**
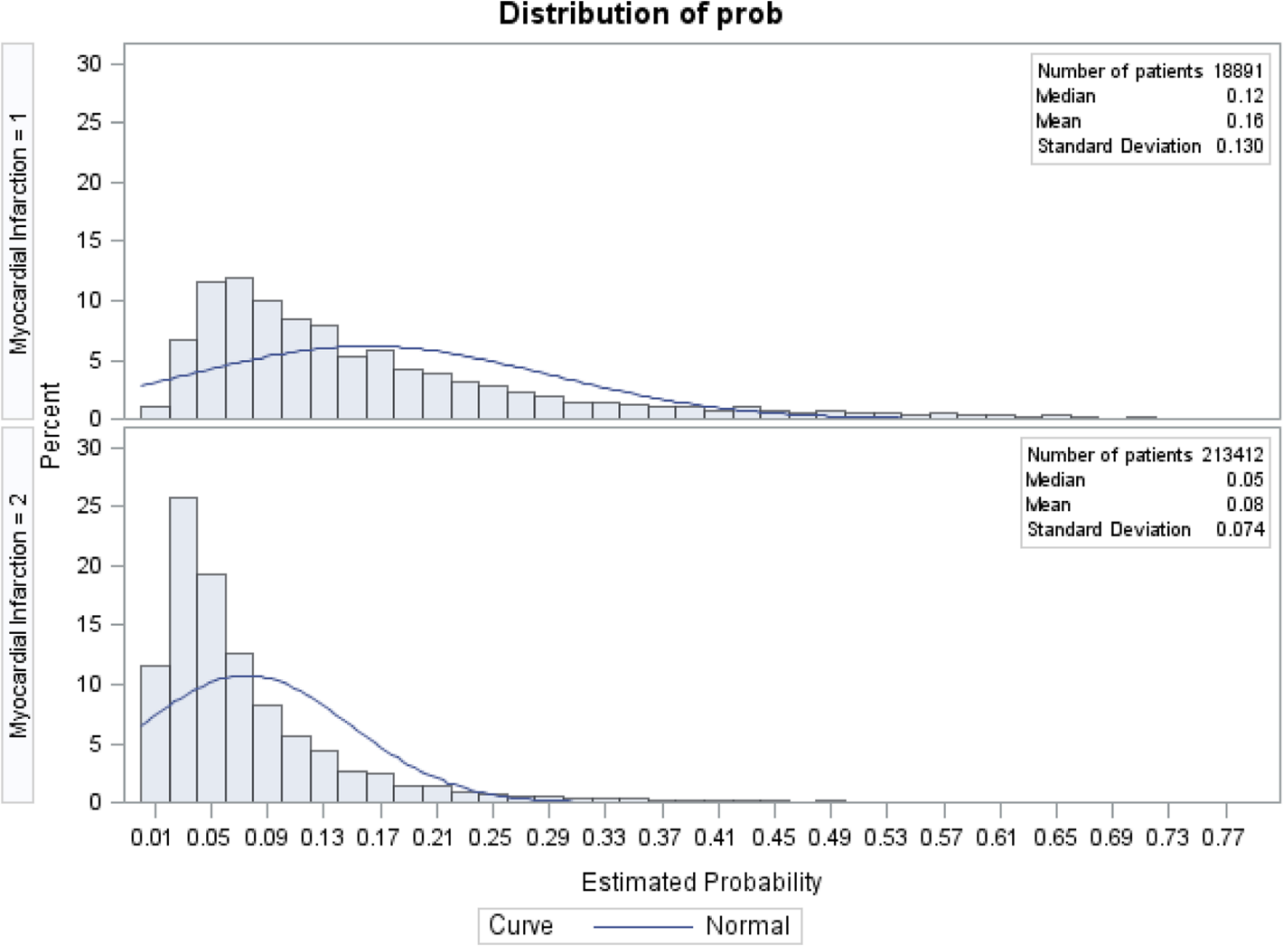
Propensity Score distribution before matching. Myocardial infarction = 1 denotes presence of myocardial infarction, whereas, Myocardial infarction = 2 denotes absence of myocardial infarction

**Fig. 2:**
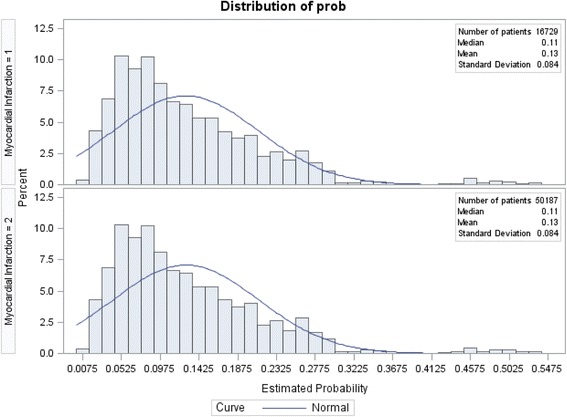
Propensity Score distribution after matching. Myocardial infarction = 1 denotes presence of myocardial infarction, whereas, Myocardial infarction = 2 denotes absence of myocardial infarction

Prior to matching, there were 18,891 MI survivors meeting inclusion criteria and 213,412 non-MI controls. The final sample, after matching, consisted of 16,729 MI survivors matched to 50,187 controls (*n* = 66,916). Table 1 describes MI survivors and non-MI controls by individual-level characteristics prior to and after matching. The majority of survivors were aged 65 years and older (61.7%), white (76.9%), unemployed (81.2%), and had insurance (95.2%). Additionally, the majority had a usual source of care (94.8%). Propensity score distributions in Figs. 1 and 2 show the balance between the two groups after propensity score matching.

**Table 1:** Overall Pre-Matching versus Post-matching Sample Description

	Before matching			After matching		
	MI	Non-MI		MI	Non-MI	
	Wt%	Wt%	*p*-value	Wt%	Wt%	*p*-value
Characteristics						
Age			<0.0001			0.5364
50–64	38.29	58.26		39.35	38.78	
65 and older	61.71	41.74		60.65	61.22	
Gender			<0.0001			0.8196
Female	37	55.63		38.01	37.82	
Male	63	44.37		61.99	62.18	
Race/ethnicity			0.0007			0.1054
White	76.94	74.46		77.48	76.02	
Non-White	23.06	25.54		22.52	23.98	
BMI			<0.0001			0.6934
Normal weight/underweight	24.77	30.61		25.01	24.62	
Overweight	37.84	38.59		38.4	38.05	
Obese	37.4	30.8		36.6	37.34	
Smoking Status			<0.0001			0.6299
Current Smoker	19.26	13.51		17.51	18.11	
Former Smoker	46.47	33.79		46.25	46.27	
Never Smoker	34.27	52.7		36.24	35.62	
Marital Status			<0.0001			<0.001
Married	53.61	62.37		54.64	60.21	
Widowed	20.81	13.98		20.7	16.57	
Sep/Divorced	20.52	16.52		19.73	16.72	
Never Married	5.06	7.13		4.93	6.5	
Education			<0.0001			<0.0001
< HS	23.55	13.37		22.17	16.3	
HS Grad	32.5	28.87		32.63	30.72	
Some College/TS	29.05	30.73		29.55	30.48	
College/TS Grad	14.9	27.03		15.65	22.5	
Employment Status			<0.0001			<0.0001
Employed	18.76	43.41		20.6	29.05	
Unemployed	81.24	56.69		79.4	70.95	
Family Income			<0.0001			<0.0001
< $25,000	34	21.9		34.72	26.45	
$25,000 - $35,000	10.55	9.18		10.88	10.52	
$35,000 - $50,000	13.13	12.04		13.7	12.91	
$50,000 - $75,000	10.42	13.34		11.37	13.95	
> $75,000	12.87	27.26		14.14	21.74	
Missing/Don’t Know	17.02	16.29		15.18	14.42	
Insurance Status			<0.0001			0.342
Yes Insurance	95.2	94.22		95.07	95.56	
No Insurance	4.8	5.78		4.93	4.44	
Usual Source of Care			<0.0001			0.1174
Yes	94.75	90.78		94.79	94.02	
No	5.23	9.22		5.21	5.98	
Region of the U.S.			<0.0001			0.0152
Northeast	18.44	18.96		18.25	17.79	
Midwest	22.93	22.62		23.23	23.1	
South	41.05	36.49		40.61	38.91	
West	17.58	21.94		17.91	20.21	
Metro Status			<0.0001			<0.0001
Metro	76.44	81.73		76.37	79.96	
Non-metro	23.56	18.27		23.63	20.04	

Note: Sample before matching: *n* = 18,891 (MI survivors), *n* = 213,412 (controls); Sample after matching: *n* = 16,729 (MI survivors), *n* = 50,187 (controls); Sample matched on the following characteristics: age, gender, race and ethnicity, smoking status, BMI, and comorbidities (cancer, arthritis, stroke, diabetes, hypertension, depression, and asthma); Significance set at *p* < 0.05

*Abbreviations: MI* myocardial infarction, *Wt%* Weight percent

HRQoL outcomes between survivors and non-MI controls based on univariate analysis are described in Table 2. Before and after matching, the majority of MI survivors rated their general health as lower compared to controls (51.2% vs. 21.4% before matching and 48.4% vs. 29.6% after matching). MI survivors also reported more limitation in daily activities (51.2% vs. 27.3% before matching and 48.4% vs. 36.8% after matching). Both MI survivors and matched controls reported a lack of emotional support (78.1 and 80.9%, respectively). Among MI survivors, 28.2% reported negative physical health and only 13.8% reported negative mental health for greater than 15 days out of the month. Among matched controls, 12.1% reported negative physical health for more than 15 days a month and 7.2% reported negative mental health for more than 15 days a month.

**Table 2:** Comparison of health-related quality of life domains between survivors of myocardial infarction and controls

	Before matching			After matching		
	MI	Non-MI	*p*-value	MI	Non-MI	*p*-value
	Wt%	Wt%		Wt%	Wt%	
Perceived general health			<0.0001			<0.001
Excellent/Very good	18.17	46.71		20.01	35.55	
Good	30.61	31.87		31.61	34.82	
Fair/poor	51.23	21.42		48.38	29.62	
Received needed emotional support			<0.001			0.1263
Always/Usually	12.47	6.78		12.19	8.94	
Sometimes	10.68	9.55		9.76	10.19	
Rarely/Never	76.86	83.67		78.05	80.88	
Activity Limitations			<0.0001			<0.0001
Yes	51.15	27.32		48.41	36.81	
No	48.85	72.68		51.6	63.2	
Life Satisfaction			<0.0001			0.024
Very satisfied/Satisfied	91.81	95.71		92.22	94.95	
Dissatisfied/Very dissatisfied	8.19	4.29		7.78	5.05	
Received recommended sleep			0.001			0.1071
Yes	47.74	55.27		48.79	53.58	
No	52.26	44.73		51.2	46.42	
Days mental health not good			<0.0001			<0.0001
None	66.5	72.64		68.58	71.45	
1–15	19.67	20.11		19.19	19.34	
> 15	13.82	7.24		12.23	9.21	
Days physical health not good			<0.0001			<0.0001
None	42.58	62.68		45.27	55.78	
1–15	29.23	25.18		29.32	27.29	
> 15	28.2	12.13		25.41	16.93	

Note: Sample before matching: *n* = 18,891 (MI survivors), *n* = 213,412 (controls); Sample after matching: *n* = 16,729 (MI survivors), *n* = 50,187 (controls); Significance set at *p* < 0.05

*Abbreviations: MI* myocardial infarction, *Wt%* Weighted percent

Adjusted odds ratios for HRQoL outcomes between survivors and non-MI controls are shown in Table 3. Survivors were approximately 2.7 times more likely to report fair/poor general health compared to controls (AOR = 2.72, 95% CI: 2.43–3.05) and approximately 1.5 times more likely to report limitations to daily activities (AOR = 1.46, 95% CI: 1.34–1.59). Survivors were more likely to report poor physical health >15 days in the month (AOR = 1.63, 95% CI: 1.46–1.83) and poor mental health >15 days in the month (AOR = 1.25, 95% CI: 1.07–1.46) compared to controls. There was no difference in MI survivors compared to controls in level of emotional support (rarely/never: AOR = 0.75, 95% CI: 0.48–1.18; sometimes: AOR = 0.73, 95% CI: 0.41–1.28), hours of recommended sleep (AOR = 1.14, 95% CI: 0.94–1.38), or life satisfaction (AOR = 1.62, 95% CI: 0.99–2.63).

**Table 3:** Adjusted odds ratios and 95% confidence intervals for survivors of MI versus controls from binary and multinomial logistic regressions on health-related quality of life

	AOR	95% CI	*p*-value
Perceived general health			
Fair/poor	2.72	[2.43,3.05]	<0.0001
Good	1.57	[1.41,1.75]	<0.0001
Excellent/Very good	Ref		
Received needed emotional support			
Rarely/never	0.75	[0.48,1.18]	0.2178
Sometimes	0.73	[0.41, 1.28]	0.2713
Always/Usually	Ref		
Activity limitations			
Yes	1.46	[1.34,1.59]	<0.0001
No	Ref		
Life Satisfaction			
Very satisfied/Satisfied	1.62	[0.99,2.63]	0.0531
Dissatisfied/very dissatisfied	Ref		
Received hours of recommended sleep			
Yes	1.14	[0.94,1.38]	0.1755
No	Ref		
Days mental health not good			
1–15	1.06	[0.96,1.18]	0.2761
> 15	1.25	[1.07,1.46]	0.0049
None	Ref		
Days physical health not good			
1–15	1.28	[1.16,1.41]	<0.0001
> 15	1.63	[1.46,1.83]	<0.0001
None	Ref		

Note: Reference categories for dependent variables are: perceived general health (excellent/very good), received needed emotional support (usually/always), activity limitations (no), life satisfaction (very dissatisfied/satisfied), received hours of recommended sleep (no), days mental health not good (0 days), days physical health not good (0 days)

Sample size after matching: *n* = 16,729 (MI survivors), *n* = 50,187 (controls); Significance set at *p* < 0.05

For all dependent variables, with the exception of the emotional support, life satisfaction, and required sleep, missing data among the study sample was less than 5%. The dependent variables on emotional support, life satisfaction and required sleep are derived from the optional modules on “emotional support and life satisfaction” and “anxiety and depression” respectively, which may have resulted in higher missingness of these variables.

## Discussion

Findings from this study demonstrate that MI survivors experience worse HRQoL compared to the general population on domains of general health, daily activity, physical health, and mental health in the U.S. This disparity remained after controlling for patient characteristics that are known predictors of reduced HRQoL after MI. No differences were noted in terms of emotional support, life satisfaction, or recommended sleep after propensity score matching.

Findings from this study are consistent with studies that showed the HRQoL most affected after experiencing MI were related to physical and general health [14, 15]. Specifically, Failde and colleagues found a decrease in the dimensions of physical functioning, general health, and vitality 3 months after acute coronary syndrome (ACS) among a Spanish population [14]. A study by Ecochard and colleagues in 2001 found that individuals perceived an inferior quality of life and impaired physical mobility 1 year post MI among French citizens [15]. Both studies observed differences in HRQoL related to older age or female sex. Our study matched case and control groups on variables including sex and age, which have been shown to be important factors in HRQoL. To our knowledge, no study comparing MI survivors to the general population with similar findings have been conducted in the U.S.

Rancic et al. [28] found a decrease in HRQoL 1 month after first MI in the domains of mobility, usual daily activities, and anxiety/depression of the EQ-5D in a Canadian population [28]. Another study among a German population found a significant reduction in HRQoL among MI survivors compared to the general population on domains of usual activities and anxiety/depression [3]. Depression has been shown to be common following MI and a predictor of reduced HRQoL on physical dimensions [29, 30]. One prospective study found that depression and anxiety had the highest impact compared to other factors on HRQoL among an Austrian population [31]. In our study, MI survivors were matched against controls on comorbid depression. Despite this, MI survivors were still more likely than controls to report decreased mental health more than 15 days of the preceding month. The American Academy of Family Physicians (AAFP) currently recommends mental health screenings post-MI to improve cardiovascular outcomes [29]. Access to mental health screenings and mental health services in this population may also help to improve HRQoL on the mental health domain.

Differences in emotional support, recommended sleep, and life satisfaction were not observed between MI survivors and the general population after matching. Literature shows that social support has a role in recovery after MI and that perceived lack of social support has an impact on HRQoL. Bosworth et al. found that individual characteristics such as age, sex, and severity of disease modified this effect [32]. Our study likely differed from these findings because we controlled for characteristics that may influence HRQoL. Although sleep disturbance among MI survivors has been shown to impact HRQoL [33], our study did not identify a difference between MI survivors and controls after matching. Additional studies identified associations between sleep disturbance, depression, managing daily activities and fatigue in patients who had experienced an MI [34, 35]. The degree to which other characteristics influence individual HRQoL on these domains warrants further investigation.

Several sociodemographic variables have been identified as predictors of reduced HRQoL in prior literature. Some of these factors include: marital status [36], employment status [17, 37], socioeconomic status [38, 39], education level [13, 37, 40], and existing psychosocial issues such as depression or anxiety [31, 41]. In our study, propensity score matching was performed on co-morbid depression in addition to other co-morbidities and the final regression model was adjusted for additional factors (marital status, employment status, income, and level of education) to remove residual bias. Future research on the extent of influence of these additional factors may be useful to identify additional risk factors for persistently low HRQoL after MI. Findings from this study suggest that MI survivors may benefit from interventions that focus on improving physical functioning and activities of daily living.

One of the strengths of this study was using propensity scores to match cases to controls using a recent, large sample of survey data. To provide more rigorous control of confounding bias, propensity score matching was used to match cases to controls on individual factors known to affect cardiovascular risk. Additionally, prior studies were conducted in an international setting and generalization to a U.S. population is limited. By utilizing national survey data obtained from a U.S. sample, our study provides data that is more representative of MI survivors within the U.S. Since this study relied on self-reported data, it is subject to response bias. Prior research has demonstrated that concordance between patient self-report and chart-based assessments varies by disease state [42]. Specifically, condition data for diagnoses such as hypertension, stroke, and diabetes has shown higher concordance than other conditions such as heart failure or arthritis. Furthermore, Corser et al. [42] found approximately 86% concordance between medical record documentation and self-report among ACS patients demonstrating that even though the overall percent agreement is high, survey comorbidity data may not be entirely concordant for this population. As such, patient self-report of comorbidities in this study may be limiting. Time since MI is unknown and there is conflicting research on how long differences in HRQoL may persist over time [3, 28, 43, 44]. Specifically, improvement in HRQoL domains over a 2 year period has been demonstrated in prior literature with the greatest improvement seen within the first 6 months [45]. Given the cross-sectional nature of BRFSS, it is unknown how HRQoL outcomes may have varied over time in this population and may have impacted study results, limiting interpretability. Future research investigating changes in HRQoL over time among a nationally representative sample may be warranted to determine how focuses on improvement may change with early versus late health statuses. It was also unknown if participants underwent cardiac rehabilitation therapy after their MI, which has been shown to significantly improve HRQoL [46]. Type of treatment method for MI has also been shown to impact HRQoL [28, 45]. Since the BRFSS does not include medication data or treatment method for MI, such as complete revascularization, it is impossible to know how this factor may have influenced our results. Additional factors such as prior percutaneous coronary intervention (PCI) or coronary artery bypass graft (CABG) were unknown and may have influenced our findings. Additionally, it was impossible to capture the HRQoL of survivors who died and it is unknown how results of this study would have been impacted by including them in the study.

Among the study sample, data were missing for less than 5% for the majority of the dependent variables. The BRFFS utilizes respondent-level statistical weighting to adjust for non-response bias; this weighting is a generally accepted practice in health survey research. Research using multiple imputation methods with BRFSS data have shown improved national level estimates [47]. Thus, it is possible that the level of emotional support, life satisfaction and required sleep among MI survivors may be underestimated. Furthermore, prior literature utilizing similar methodology with BRFSS data for estimates of HRQoL have not focused on level of missingness among the large sample of data or have excluded respondents with missing data on any outcome [26].

## Conclusion

MI survivors experienced lower HRQoL on domains of general health, physical health, and mental health compared to the general population when controlling for some known predictors of reduced HRQoL after myocardial infarction. There were no differences in MI survivors and the general population on domains of recommended sleep, emotional support, or life satisfaction. Clinical interventions focused on physical functioning, activities of daily living, and improving access to mental health services may improve HRQoL among MI survivors in the U.S. Further research examining factors contributing to reduced HRQoL on domains of general health, physical health, and mental health is warranted.

## Abbreviations

AOR: Adjusted Odds Ratio
BMI: Body mass index
BRFSS: Behavioral Risk Factor Surveillance System
CABG: Coronary Artery Bypass Graft
CI: Confidence Intervals
HRQoL: Health-Related Quality of Life
MI: Myocardial infarction
OR: Odds Ratio
PCI: Percutaneous Coronary Intervention
US: United States
